# Urban–rural disparities in diabetes-related mortality in the USA 1999–2019

**DOI:** 10.1007/s00125-022-05785-4

**Published:** 2022-09-10

**Authors:** Ofer Kobo, Harriette G. C. Van Spall, Mamas A. Mamas

**Affiliations:** 1 Department of Cardiology, Hillel Yaffe Medical Centre, Hadera, Israel; 2 Keele Cardiovascular Research Group, Centre for Prognosis Research, Institute for Primary Care and Health Sciences, Keele University, Keele, UK; 3 Department of Medicine, McMaster University, Hamilton, ON Canada; 4 Department of Health Research Methods, Evidence, and Impact, McMaster University, Hamilton, ON Canada; 5 Population Health Research Institute, Hamilton, ON Canada; 6 Research Institute of St Joseph’s, Hamilton, ON Canada; 7 Department of Cardiology, Thomas Jefferson University, Philadelphia, PA USA; 8 Institute of Population Health, University of Manchester, Manchester, UK

**Keywords:** Diabetes-related mortality, Disparities, Epidemiology, Prognosis, Rural–urban mortality gap

## Abstract

**Aims/hypothesis:**

Our study aimed to examine the trends in diabetes-related mortality in urban and rural areas in the USA over the past two decades.

**Methods:**

We examined the trends in diabetes-related mortality (as the underlying or a contributing cause of death) in urban and rural areas in the USA between 1999 and 2019, using the CDC WONDER Multiple Cause of Death database. We estimated the 20 year trends of the age-adjusted mortality rate (AAMR) per 100,000 population in urban vs rural counties.

**Results:**

The AAMR of diabetes was higher in rural than urban areas across all subgroups. In urban areas, there was a significant decrease in the AAMR of diabetes as the underlying (−16.7%) and contributing (−13.5%) cause of death (*p*_trend_<0.001), which was not observed in rural areas (+2.6%, +8.9%, respectively). AAMRs of diabetes decreased more significantly in female compared with male individuals, both in rural and urban areas. Among people younger than 55 years old, there was a temporal increase in diabetes-related AAMR (+13.8% to +65.2%). While the diabetes-related AAMRs of American Indian patients decreased in all areas (−19.8% to −40.5%, all *p*_trend_<0.001), diabetes-related AAMRs of Black and White patients decreased significantly in urban (−26.6% to −28.3% and −10.7% to −15.4%, respectively, all *p*_trend_<0.001) but not rural areas (−6.5% to +1.8%, +2.4% to +10.6%, respectively, *p*_trend_ NS, NS, NS and <0.001).

**Conclusions/interpretation:**

The temporal decrease in diabetes-related mortality in the USA has been observed only in urban areas, and mainly among female and older patients. A synchronised effort is needed to improve cardiovascular health indices and healthcare access in rural areas and to decrease diabetes-related mortality.

**Graphical abstract:**

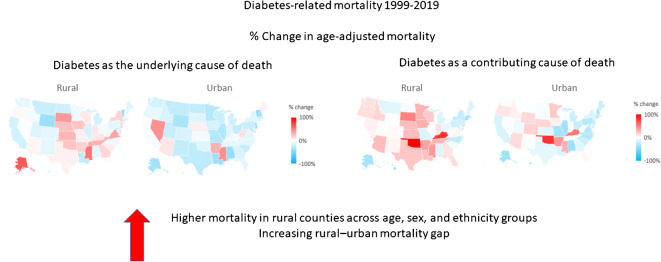

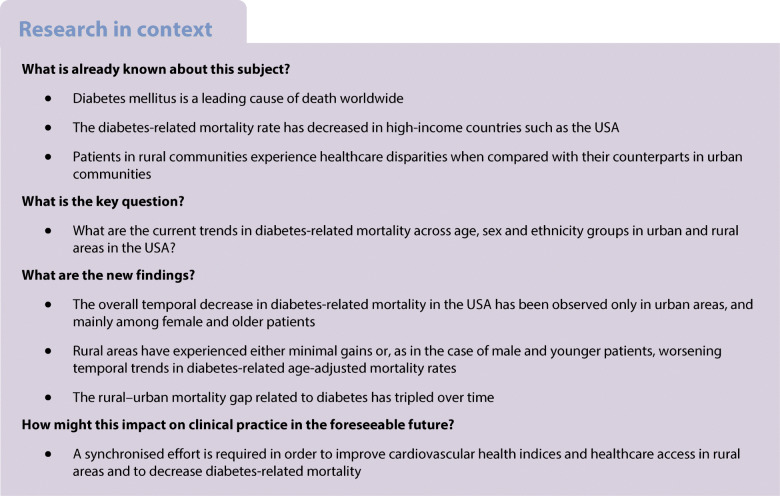



## Introduction

Diabetes mellitus is a leading cause of mortality worldwide. While the diabetes-related mortality rate has decreased in high-income countries such as the USA [1], these trends may not apply to all regions. Rural populations, including older adults and individuals of ethnic minority descent, may be at an increased risk of developing type 2 diabetes [2]. When compared with counterparts in urban communities, patients in rural communities may also experience healthcare disparities that translate to urban–rural differences in diabetes-related mortality. We therefore examined the trends in diabetes-related mortality across age, sex and ethnicity groups in urban and rural areas in the USA over the past two decades.

## Methods

We used the Centers for Disease Control and Prevention Wide-ranging ONline Data for Epidemiologic Research (CDC WONDER) Multiple Cause of Death database [3]. Data were based on death certificates for U.S. residents over the period 1999–2019. Each death certificate contained a single underlying cause of death, up to 20 additional multiple causes and demographic data including ethnicity [3]. Race and Hispanic origin are reported separately on the death certificate in accordance with standards set forth by the Office of Management and Budget. Therefore, as Hispanic origin was not reported on the death certificate for some deaths, we did not report Hispanic origin in this analysis. The study was exempt from Institutional Review Board approval because the CDC WONDER Multiple Cause of Death database contains anonymised, publicly available data. Diabetes-related mortality was identified using the ICD-10 (http://apps.who.int/classifications/icd10/browse/2016/en) codes E10-E14 either as an underlying or as a contributing cause of death. The population was divided into urban (large, medium and small metropolitan areas) and rural (non-metropolitan areas) counties according to the 2013 U.S. Census classification, which is based on the Office of Management and Budget’s February 2013 delineation of metropolitan statistical areas and micropolitan statistical areas [4]. Crude mortality rates and age-adjusted mortality rates (AAMRs) are presented per 100,000 population. Mortality rates were calculated for each year. The population estimates used as the denominators of rates were race-, sex- and age-specific and were obtained each year for the rural and urban population from the CDC WONDER website. Beginning with the 1999 data year, the National Center for Health Statistics adopted the age distribution of the year 2000 population of the USA as the standard population for the purpose of age adjustments. The AAMRs are provided by the CDC WONDER website and were calculated yearly using the direct standardisation method based on the age group weights from the 2000 U.S. population. We presented the per cent change in crude mortality rates and AAMRs between two time points (1999 and 2019), and identified trends in AAMRs between 1999 and 2019 using the Pearson correlation trend test and its *p* value using yearly data points. Statistical significance was set at the *p*<0.05 level.

## Results

Between 1999 and 2019 we identified 1,572,536 death certificates with diabetes mellitus as the underlying cause of death (of which 79.8% were in urban counties), and 5,025,745 death certificates with diabetes mellitus as a contributing cause of death (of which 79.5% were in urban counties).

**Fig. 1:**
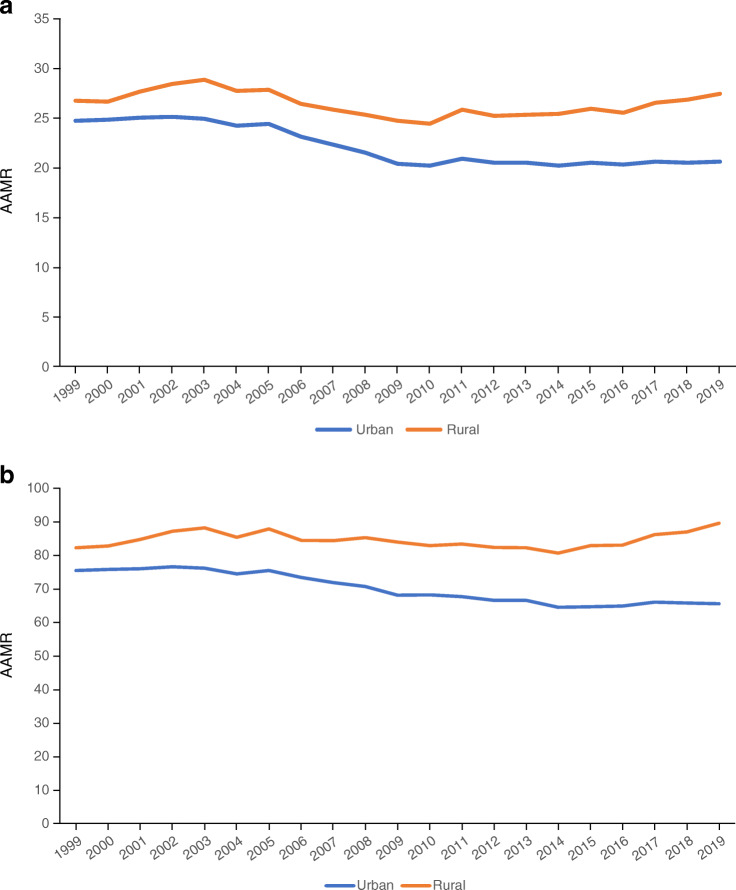
Trends in age-adjusted diabetes-related mortality in the USA 1999–2019, stratified by urbanisation status, per 100,000 population. Diabetes as the underlying cause of death (**a**); and diabetes as a contributing cause of death (**b**)

The AAMR of diabetes was higher in rural than urban areas across age, sex and ethnicity groups (Table 1). Figure 1 presents the trends in AAMR of diabetes between 1999 and 2019. In urban areas, there was a significant decrease in the AAMR of diabetes as the underlying (−16.7%) and contributing (−13.5%) cause of death (*p*_trend_<0.001 for both) in the period 1999–2019. In rural areas, there was no significant temporal change in the AAMR of diabetes as the underlying (+2.6%) or contributing (+8.9%) cause of death (both *p*_trend_ not significant). The rural–urban difference in AAMR increased approximately threefold (from 2.0 to 6.8 and from 6.8 to 24.3 for diabetes as the underlying and contributing cause of death, respectively). In both rural and urban areas, AAMRs of diabetes as the underlying and contributing cause of death were higher in male than female individuals. Furthermore, as the AAMRs of diabetes decreased more significantly in female compared with male individuals, both in rural and urban areas during the study period, the male–female diabetes mortality gap widened over time in rural and urban areas for diabetes as the underlying and contributing cause of death (*p*_trend_<0.05, Table 1).

**Table 1:** Differences in urban and rural mortality rates related to diabetes, 1999–2019

Characteristic	Total number of deaths			Crude mortality rate^a^ (95% CI)			AAMR^a^ (95% CI)		
	1999	2019	% Change	1999	2019	% Change	1999	2019	% Change
Diabetes as the underlying cause of death									
Urban areas	54,747	69,848	+27.6	23.4 (23.2, 23.5)	24.8 (24.6, 24.9)	+6.0	24.6 (24.4, 24.9)	20.5 (20.4, 20.7)	−16.7
Female	29,711	30,313	+2	24.8 (24.5, 25.1)	21.1 (20.9, 21.3)	−14.9	22.6 (22.3, 22.8)	15.9 (15.7, 16.1)	−29.6
Male	25,036	39,535	+57.9	21.8 (21.5, 22.1)	28.5 (28.2, 28.8)	+30.7	27.5 (27.1, 27.8)	26.2 (25.9, 26.4)	−4.7
Under 55	6147	7877	+28.1	3.3 (3.2, 3.4)	3.9 (3.8, 4.0)	+18.2	3.4 (3.3, 3.5)	3.9 (3.8, 4.0)	+14.7
55 and over	48,597	61,968	+27.5	102.1 (101.2, 103.0)	76.7 (76.1, 77.3)	−24.9	102.9 (102.0, 103.8)	81.7 (81.0, 82.3)	−20.6
American Indian^b^	334	585	+75.1	17.8 (15.9, 19.7)	16.9 (15.5, 18.2)	−5.1	38.1 (33.7, 42.6)	22.7 (20.7, 24.6)	−40.5
Asian^c^	1104	3301	+199	10.0 (9.4, 10.6)	15.6 (15.1, 16.2)	+56.0	18.3 (17.2, 19.4)	16.4 (15.8, 16.9)	−10.4
Black^d^	10,446	13,626	+30.4	32.3 (31.7, 32.9)	32.1 (31.5, 32.6)	−0.6	49.8 (48.8, 50.8)	35.7 (35.0, 36.3)	−28.3
White	42,863	52,336	+22.1	22.7 (22.4, 22.9)	24.3 (24.1, 24.5)	+7	22.1 (21.9, 22.3)	18.7 (18.6, 18.9)	−15.4
Rural areas	13,652	17,799	+30.4	30.6 (30.1, 31.1)	38.6 (38.1, 39.2)	+26.1	26.6 (26.2, 27.1)	27.3 (26.9, 27.1)	+2.6
Female	7538	7822	+3.8	33.4 (32.7, 34.2)	34.0 (33.3, 34.8)	+1.8	24.8 (24.3, 25.4)	22.0 (21.5, 22.5)	−11.3
Male	6114	9977	+63.2	27.7 (27.0, 28.4)	43.2 (42.4, 44.1)	+56.0	29.0 (28.2, 29.7)	33.5 (32.8, 34.2)	+15.5
Under 55	1309	1862	+42.2	3.9 (3.7, 4.1)	6.1 (5.9, 6.4)	+56.4	3.9 (3.6, 4.1)	6.2 (5.9, 6.5)	+59.0
55 and over	12,343	15,937	+29.1	112.4 (110.4, 114.4)	101.4 (99.8, 103.0)	−9.8	110.5 (108.5, 112.4)	105.2 (103.6, 106.8)	−4.8
American Indian^b^	391	565	+44.5	40.9 (36.8, 44.9)	43.0 (39.5, 46.6)	+5.1	80.5 (72.2, 88.9)	50.2 (45.9, 54.4)	−37.6
Asian^c^	44	134	+204.5	12.6 (9.2, 16.9)	19.2 (16.0, 22.5)	+52.4	19.6 (14.1, 26.4)	20.4 (16.9, 23.9)	+4.1
Black^d^	1481	2067	+39.6	38.9 (36.9, 40.9)	50.3 (48.1, 52.4)	+29.3	49.4 (46.9, 52.0)	50.3 (48.1, 52.5)	+1.8
White	11,736	15,033	+28.1	29.7 (29.2, 30.2)	37.6 (37.0, 38.2)	+26.6	24.6 (24.2, 25.1)	25.2 (24.8, 25.7)	+2.4
Diabetes as a contributing cause of death									
Urban areas	167,277	223,381	+33.5	71.4 (71.0, 71.7)	79.2 (78.8, 79.5)	+10.9	75.4 (75.1, 75.8)	65.2 (65.0, 65.7)	−13.5
Female	87,846	98,692	+12.3	73.4 (72.9, 73.9)	68.7 (68.3, 69.2)	−6.4	66.4 (65.9, 66.8)	51.1 (50.8, 51.4)	−23.0
Male	79,431	124,689	+57.0	69.2 (68.7, 69.7)	90.0 (89.5, 90.5)	+30.1	88.3 (87.7, 88.9)	83.5 (83.0, 84.0)	−5.4
Under 55	14,565	18,303	+25.7	7.8 (7.7, 7.9)	9.1 (9.0, 9.2)	+16.7	8.0 (7.9, 8.2)	9.1 (9.0, 9.2)	+13.8
55 and over	152,705	205,062	+34.3	320.8 (319.2, 322.4)	253.8 (252.7, 254.9)	−20.9	323.5 (321.9, 325.1)	272.9 (271.7, 274.1)	−15.6
American Indian^b^	760	1693	+122.8	40.5 (37.6, 43.4)	48.8 (64.4, 51.1)	+20.5	89.8 (82.9, 96.8)	67.0 (63.7, 70.4)	−25.4
Asian^c^	3655	9934	+171.8	33.2 (32.2, 34.3)	47.0 (46.1, 48.0)	+41.6	61.0 (58.9, 63.1)	49.8 (48.8, 50.8)	−18.4
Black^d^	27,961	37,231	+33.2	86.4 (85.4, 87.4)	87.6 (86.7, 88.5)	+1.4	134.2 (132.6, 135.8)	98.5 (97.5, 99.6)	−26.6
White	134,901	174,523	+29.4	71.3 (70.9, 71.7)	81.1 (80.8, 81.5)	+13.7	69.4 (69.1, 69.8)	62.0 (61.7, 62.3)	−10.7
Rural areas	42,387	59,420	+40.2	95.0 (94.1, 95.9)	129.0 (128.0, 130.0)	+35.8	82.2 (81.4, 83.0)	89.5 (88.8, 90.2)	+8.9
Female	22,938	26,304	+14.7	101.7 (100.4, 103.0)	114.4 (113.0, 115.8)	+12.5	74.7 (73.7, 75.7)	72.0 (71.1, 72.9)	−3.6
Male	19,449	33,116	+70.3	88.2 (86.9, 89.4)	143.5 (142.0, 145.1)	+62.7	92.6 (91.3, 94.0)	110.3 (109.1, 111.5)	+19.1
Under 55	3040	4461	+46.7	9.0 (8.7, 9.4)	14.7 (14.3, 15.1)	+63.3	8.9 (8.6, 9.2)	14.7 (14.3, 15.1)	+65.2
55 and over	39,347	54,959	+39.7	358.4 (354.8, 361.9)	349.8 (346.8, 352.7)	−2.4	351.9 (348.4, 355.3)	364.9 (361.8, 367.9)	+3.7
American Indian^b^	831	1561	+87.8	86.9 (81.0, 92.8)	118.8 (112.9, 124.7)	+36.7	172.2 (159.9, 184.5)	138.1 (131.0, 145.1)	−19.8
Asian^c^	196	380	+93.9	56.2 (48.3, 64.1)	54.5 (49.1, 60.0)	−3.0	94.4 (80.9, 108.0)	58.0 (52.1, 63.9)	−38.6
Black^d^	4114	5288	+28.5	108.1 (104.8, 111.4)	128.6 (125.1, 132.0)	+19	137.6 (133.4, 141.8)	128.7 (125.2, 132.3)	−6.5
White	37,246	52,191	+40.1	94.3 (93.3, 95.2)	130.7 (129.6, 131.8)	+38.6	77.6 (76.8, 78.4)	85.8 (85.1, 86.6)	+10.6

^a^Mortality rates per 100,000 people

^b^American Indian or Alaska Native

^c^Asian or Pacific Islander

^d^Black or African American

Among people younger than 55 years old, there was a temporal increase in diabetes-associated AAMR; this increase was larger in rural vs urban areas for diabetes as both the underlying (+59.0% rural, +14.7% urban, both *p*_trend_<0.001) and contributing (+65.2% rural, +13.8% urban, both *p*_trend_<0.001) cause of death. In older patients there was a large decrease in diabetes-related AAMR in urban areas (−20.6% and −15.6% for the underlying and contributing cause of death, respectively, both *p*_trend_<0.001) but not in rural areas (−4.8% and +3.7% for the underlying and contributing cause of death, respectively, both *p*_trend_ NS).

American Indian and Black patients had substantially higher diabetes-related AAMRs than Asian and White patients. Within each ethnic group, AAMR was higher in rural areas. While the diabetes-related AAMRs of American Indian patients decreased in all areas (−19.8% to 40.5%, all *p*_trend_<0.001), the diabetes AAMRs of Black patients decreased significantly in urban (−28.3% and −26.6% for the underlying and contributing cause of death, respectively, both *p*_trend_<0.001) but not rural areas (+1.8% and −6.5% for diabetes as the underlying and contributing cause of death, respectively, both *p*_trend_ NS). Similar findings were observed in White patients (−15.4% and −10.7% in urban areas, both *p*_trend_ NS; +2.4% and +10.6% in rural areas, *p*_trend_ NS, <0.001).

## Discussion

Our findings demonstrate that the overall temporal decrease in diabetes-related mortality in the USA has been observed only in urban areas and mainly among female and older patients. Rural areas have experienced either minimal gains or, as in the case of male and younger patients, worsening temporal trends in diabetes-related AAMR. The highest AAMRs have been in American Indian and Black patients residing in rural areas. The rural–urban mortality gap related to diabetes has tripled over time.

We observed increasing rates of diabetes-related mortality among younger adults over the last 20 years, compared with the older population. The increased mortality among the younger adults may be related to the increasing prevalence of type 2 diabetes in adolescents and young adults. Early-onset type 2 diabetes is associated with more aggressive disease and higher rates of premature complications [5]. Moreover, a previous study reported worse glucose control among younger adults with type 2 diabetes [6]. The fact that male individuals are more likely to be diagnosed with diabetes at an early age [7], may explain the widening male–female diabetes-related mortality gap in both urban and rural areas.

Residents of rural areas are at increased risk of diabetes as the prevalence of obesity and the metabolic syndrome is higher in rural areas [8, 9]. Furthermore, rural residents are less likely to have participated in diabetes self-management education programmes [10]. Indeed, rural patients were found to have higher rates of diabetes-related emergency department use compared with urban patients [11].

Our finding of an increasing gap in diabetes outcomes is in concordance with previous studies that reported greater improvements in blood pressure and cholesterol control for urban adults with diabetes than for those in rural areas over the last two decades. These differences remained significant even after multiple adjustments for ethnicity, education, poverty levels and clinical characteristics [12]. The management of diabetes and its complications requires expertise that may be difficult to access in rural communities. Residents of rural counties are less likely to have usual primary care provided by physicians [13]. Furthermore, there has been a disproportionate closure of hospitals in rural areas [14].

The role of socioeconomic deprivation and structural racism in the incidence of cardiovascular risk factors, progression of diabetes, and survival rates must also be considered, particularly in American Indian and Black individuals. Cardiometabolic risk varies across ethnic groups and areas, and is inextricably linked with social determinants of health, including education, economic resources, psychological stress and access to preventive healthcare [15]. Healthcare equity, expansion of Medicaid, and telemedicine initiatives that extend access to specialty care may mitigate some of the rural–urban disparities in mortality. However, the ultimate solutions may lie in economic and policy interventions that broaden our focus from treating disease to preventing it.

### Limitations

There are some limitations to note. First, we were able to examine disparities only by key sociodemographic characteristics that were available in the CDC WONDER database (e.g. age and race/ethnicity). Furthermore, the use of bridged race categories (e.g. the Asian or Pacific Islander race category includes Chinese, Filipino, Hawaiian, Japanese and Other Asian or Pacific Islanders) may lead to a heterogeneous group of patients where findings are difficult to interpret in relation to race. Second, we were not able to adjust for baseline comorbidities, nor for other important potential confounders, such as socioeconomic status, education and occupation. Third, the urbanisation status was defined by the place of death. We cannot rule out the possibility of that individuals toward the end of life chose to migrate (i.e. for social support and care by relatives).

### Conclusions

While diabetes-related mortality rates decreased in urban counties in the USA over the last two decades, they did not decrease in rural counties. We report that the rural–urban diabetes-related mortality gap has tripled in the USA during this period, mainly among male patients and those younger than 55 years old. A synchronised effort is required to improve cardiovascular health indices and healthcare access in rural areas and to decrease diabetes-related mortality.

## Abbreviations

AAMR: Age-adjusted mortality rate
CDC WONDER: Centers for Disease Control and Prevention Wide-ranging ONline Data for Epidemiologic Research

## References

[1] Lin X, Xu Y, Pan X (2020). Global, regional, and national burden and trend of diabetes in 195 countries and territories: an analysis from 1990 to 2025. Sci Rep.

[2] Towne SD, Bolin J, Ferdinand A, Nicklett EJ, Smith ML, Ory MG (2017). Assessing diabetes and factors associated with foregoing medical care among persons with diabetes: disparities facing American Indian/Alaska Native, Black, Hispanic, low income, and Southern adults in the U.S. (2011-2015). Int J Environ Res Public Health.

[3] Centers for Disease Control (2020) Multiple Cause of Death 1999-2019. CDC WONDER Online Database. Available from https://wonder.cdc.gov/. Accessed 10 March 2022

[4] Seto S, Onodera H, Kaido T (2000). Tissue factor expression in human colorectal carcinoma: correlation with hepatic metastasis and impact on prognosis. Cancer.

[5] Lascar N, Brown J, Pattison H, Barnett AH, Bailey CJ, Bellary S (2018). Type 2 diabetes in adolescents and young adults. Lancet Diabetes Endocrinol.

[6] Casagrande SS, Aviles-Santa L, Corsino L et al (2017) Hemoglobin A1c, blood pressure, and LDL-cholesterol control among Hispanic/Latino adults with diabetes: results from the Hispanic Community Health Study/Study Of Latinos (HCHS/SOL). Endocr Pract 23(10):1232–1253. 10.4158/EP171765.OR 10.4158/EP171765.OR PMC5796425 28816530

[7] Kautzky-Willer A, Harreiter J, Pacini G (2016). Sex and gender differences in risk, pathophysiology and complications of type 2 diabetes mellitus. Endocr Rev.

[8] Trivedi T, Liu J, Probst JC, Martin AB (2013). The metabolic syndrome: are rural residents at increased risk?. J Rural Health.

[9] Befort CA, Nazir N, Perri MG (2012). Prevalence of obesity among adults from rural and urban areas of the United States: findings from NHANES (2005-2008). J Rural Health.

[10] Luo H, Bell RA, Winterbauer NL (2022). Trends and rural-urban differences in participation in diabetes self-management education among adults in North Carolina: 2012-2017. J Public Health Manag Pract.

[11] Uppal TS, Chehal PK, Fernandes G (2022). Trends and variations in emergency department use associated with diabetes in the US by sociodemographic factors, 2008-2017. JAMA Netw Open.

[12] Mercado CI, McKeever Bullard K, Gregg EW, Ali MK, Saydah SH, Imperatore G (2021). Differences in U.S. rural-urban trends in diabetes ABCS, 1999-2018. Diabetes Care.

[13] Kirby JB, Yabroff KR (2020). Rural-urban differences in access to primary care: beyond the usual source of care provider. Am J Prev Med.

[14] Germack HD, Kandrack R, Martsolf GR (2019). When rural hospitals close, the physician workforce goes. Health Aff (Millwood).

[15] Van Spall HGC, Yancy CW, Ferdinand KC (2020). COVID-19 and Katrina: recalcitrant racial disparities. Eur Heart J.

